# Notärztliche Maßnahmen bei der Versorgung von Patient:innen mit akutem Koronarsyndrom: Eine vergleichende, retrospektive Beobachtungsstudie

**DOI:** 10.1007/s00063-025-01306-4

**Published:** 2025-08-01

**Authors:** Thomas Hofmann, Lena Himmelreich, Roland Kirschenlohr, Toni Fredrich, Patrick Andreas Eder, Frank Flake, Katrin Bagdahn, Jan Orendt, Melanie Reuter-Oppermann, Rolf Lefering

**Affiliations:** 1 Institut für Forschung in der Operativen Medizin (IFOM), Universität Witten/Herdecke, Witten/Herdecke, Deutschland; 2 Fachbereich Gesundheit und Soziales, HSD Hochschule Döpfer University of Applied Sciences, Potsdam, Deutschland; 3 Deutsche Gesellschaft für Rettungswissenschaften (DGRe), Aachen, Deutschland; 4 Feuerwehr Bremen, Bremen, Deutschland; 5 Landkreis Oldenburg, Wildeshausen, Deutschland; 6 DRK Rettungsdienst Mittelhessen, Marburg, Deutschland; 7 Innovationsmanagement, ZTM, Bad Kissingen, Deutschland; 8 ILS Mannheim gGmbH, Mannheim, Deutschland

**Keywords:** Notrufabfrage, Notarztquote, Notfallsanitäter, Rettungsdienst, Pyramidenprozess, Triage, Emergency physician quota, Paramedic, Emergency Medical services, Pyramid process

## Abstract

**Hintergrund:**

Die rettungsdienstliche Versorgung von Patient:innen mit akutem Koronarsyndrom (ACS) ist von hoher Relevanz, da solche Einsätze bisher eine überdurchschnittliche Beteiligung von Notärzt:innen erfordern. Ziel dieser Studie war es, die Inzidenz notärztlicher Maßnahmen im Rahmen der ACS-Versorgung zu untersuchen und Prädiktoren zu identifizieren, die eine Notwendigkeit solcher Maßnahmen bereits bei der Notrufabfrage erkennen lassen.

**Methoden:**

Es wurde eine retrospektive Beobachtungsstudie basierend auf 10.833 Rettungsdienstprotokollen aus 3 Regionen in Deutschland durchgeführt. Eingeschlossen wurden Protokolle mit (Verdachts‑)Diagnosen wie STEMI, NSTEMI, unklarem Thoraxschmerz oder kardiogenem Schock. Nach umfangreicher Datenbereinigung und Kodierung der Maßnahmen erfolgte eine deskriptive Analyse zu Ermittlung der Inzidenz von notärztlichen Maßnahmen sowie eine logistische Regression, um Prädiktoren für notärztliche Interventionen zu bestimmen.

**Ergebnisse:**

In 2,5 % der Fälle wurden Maßnahmen durchgeführt, die die Anwesenheit eines Notarztes erforderten. Signifikante Prädiktoren für notärztliche Maßnahmen waren ein nichtdokumentiertes (Odds-Ratio [OR] 2,7), ein getrübtes (OR 7,77) oder ein fehlendes Bewusstsein (OR 24,5), eine nichtdokumentierte Atmung (OR 5,13), eine Dyspnoe (OR 2,1), Zyanose (OR 4,48), Apnoe (OR 8,82) oder Kaltschweißigkeit (OR3,2).

**Schlussfolgerung:**

Die Inzidenz notärztlicher Maßnahmen bei ACS-Patient:innen ist gering. Die Ergebnisse deuten darauf hin, dass nicht alle ACS-Fälle eine notärztliche Versorgung erfordern. Jedoch sollten Patient:innen mit reduzierter Bewusstseinslage, Zyanose oder Atemstillstand weiterhin zeitnah durch Notärzt:innen behandelt werden. Prospektive Studien könnten die Versorgungssituation weiter verbessern.

**Zusatzmaterial online:**

Zusätzliche Informationen sind in der Online-Version dieses Artikels (https://doi.org/10.1007/s00063-025-01306-4) enthalten.

## Abstract (en)

**Background:**

The prehospital emergency care of patients with acute coronary syndrome (ACS) is highly relevant as such interventions have required an above-average involvement of emergency physicians to date. The aim of this study was to investigate the incidence of physicians’ interventions in the context of ACS care and to identify predictors that indicate the need for such interventions at the time of the emergency call.

**Methods:**

A retrospective observational study was conducted based on 10,833 emergency department protocols from three regions in Germany. Protocols with (suspected) diagnoses such as STEMI, NSTEMI, unclear chest pain, or cardiogenic shock were included. After extensive data cleaning and coding of interventions, a descriptive analysis was performed to determine the incidence of physicians’ measures and a logistic regression to determine predictors for physicians’ interventions.

**Results:**

Interventions requiring the presence of an emergency physician were performed in 2.5% of cases. Significant predictors for physicians’ interventions were undocumented (odds ratio [OR] 2.7), reduced (OR 7.77) or absent consciousness (OR 24.5), undocumented breathing (OR 5.13), dyspnea (OR 2.1), cyanosis (OR 4.48), apnea (OR 8.82) or cold sweats (OR 3.2).

**Conclusion:**

The incidence of physicians’ interventions in ACS patients is low. The results suggest that not all ACS cases require physicians on scene. However, patients with reduced consciousness, cyanosis or respiratory arrest should continue to be treated primarily by emergency physicians. Prospective studies could further improve prehospital care.

## Einführung

Die Versorgung von Patient:innen, die unter einem akuten Koronarsyndrom (ACS) leiden oder leiden könnten, stellt einen nicht unerheblichen Anteil der rettungsdienstlichen und notärztlichen Einsätze dar. Pro Jahr erkranken in Deutschland ca. 9,5 pro 1000 Einwohner:innen neu an einer koronaren Herzerkrankung [17]. Aktuelle Daten zeigen, dass ca. 3,22 % aller rettungsdienstlich versorgter Patient:innen in Deutschland mit der Verdachtsdiagnose ACS oder kardiogener Schock im Krankenhaus angemeldet werden, davon sind an ca. 66–81 % der Einsätze Notärzt:innen (NA) beteiligt. Somit ist die Notarztquote hier deutlich höher als im Durchschnitt über alle Einsätze von Notfallsanitäter:innen (NotSan), dort liegt sie bei 15,8 % [15]. Es ist also davon auszugehen, dass hochwertige NA-besetzte Rettungsmittel zu einem großen Teil in der Versorgung von ACS-Patient:innen ein- und gebunden sind.

Gleichzeitig steht das Rettungsdienstsystem unter Reformdruck. Neben internen Treibern für diese Veränderungen sind insbesondere die personelle und finanzielle Ressourcenknappheit gepaart mit kontinuierlich steigenden Einsatzzahlen die Ursachen für den Reformbedarf [14]. Als vielversprechender Lösungsansatz für diese Probleme gilt eine bessere Patient:innensteuerung und damit die Idee, hochwertige Ressourcen nur den Personen zur Verfügung zu stellen, die diese wirklich benötigen [20, 24]. Auf die rettungsdienstliche Versorgung angewandt bedeutet dieser Reformgedanke, dass auch die dortigen Ressourcen output- bzw. outcomeorientiert eingesetzt werden sollen und müssen.

Das nun über 10 Jahre alte Berufsbild der NotSan hatte bei der Einführung das klare Ziel, Patient:innen eigenständig zu versorgen, die bis dato durch NA versorgt wurden [8]. Über die hierzu erforderlichen eigenständigen Kompetenzen der NotSan erfolgt eine berufsgruppen-, regionsübergreifende und regelmäßig überarbeitete Konsentierung im Rahmen der Pyramidenprozesse [3, 7, 19].

Um die bereits begonnene Transformation des Rettungsdiensts zu steuern und patientensicher gestalten zu können, muss diese wissenschaftlich begleitet werden. Gleichwohl sind die damit verbundenen Fragestellungen so komplex und facettenreich, dass wohl kaum eine andere Möglichkeit besteht, als jede bisher überwiegend historisch gewachsene NA-Indikation hinsichtlich ihrer Auswirkung auf das Versorgungsoutput empirisch zu überprüfen.

Da die NA-Beteiligung bei Einsätzen, die mit einer ACS-Versorgung einhergehen, überdurchschnittlich hoch ist widmet sich diese Studie diesem Thema mit dem Fokus auf die durchgeführten therapeutischen Maßnahmen. Konkret soll diese Untersuchung die Fragen „Wie hoch ist die Inzidenz von notärztlichen Maßnahmen im Rahmen der Versorgung von Patient:innen mit ACS-Symptomatik?“ und „Kann die Notwendigkeit einer notärztlichen Maßnahme bei der Notrufabfrage erkannt werden?“ beantworten.

## Methoden

### Studiendesign

Basierend auf Daten aus Rettungs- und Notarztdienstprotokollen wurden die ergriffenen Maßnahmen im Rahmen der Versorgung von ACS-Patient:innen erfasst. Im Anschluss erfolgt anhand des Pyramidenprozesses die Einteilung, ob diese Maßnahmen hätten durch NotSan durchgeführt werden können. In der Folge wurde eine logistische Regression durchgeführt, um zu prüfen, ob sich Patient:innen mit notärztlichem Interventionsbedarf anhand der in den Protokollen dokumentierten Erstbefunde vorhersagen lassen. Somit wurde eine retrospektive Datenanalyse von digital erhobenen Routinedaten durchgeführt.

### Rekrutierung der Forschungsdaten

Um die Forschungsfragen beantworten zu können, stellten das DRK Rettungsdienst Mittelhessen (RDMH), der Landkreis Oldenburg (LO) und die Stadt Bremen (HB) entsprechende Protokolldaten von Rettungsdienst- und NA-Einsätzen (RDMH und LO: NIDA-System, Notfallinformations- und Dokumentationsassistent der Fa. medDV GmbH, Pohlheim, Deutschland; HB: CKS von Johnson Controls, Cork, Irland) zur Verfügung. Die geografischen und strukturellen Unterschiede der jeweiligen Regionen sollen zu einer besseren Generalisierbarkeit der Ergebnisse beitragen.

Protokolle wurden zur Verfügung gestellt und damit eingeschlossen, wenn die primäre bzw. sekundäre (Verdachts‑) Diagnose oder die IVENA-Anmeldung STEMI, NSTEMI, unklarer Thoraxschmerz oder kardiogener Schock in den digitalen Protokollen lautete. Die genauen Zeiträume der eingeschlossenen Protokolle variierten von Region zu Region, abhängig von der Einführung der digitalen Datenerfassung. Die gelieferten Datensätze umfassen Einsatzdatum, Rettungsmittel, Erstbefunde des Bewusstseinszustands, Atmung, Haut, Schmerzen und Psyche, verabreichte Medikamente und durchgeführte invasive Maßnahmen.

### Datenbereinigung

**Abb. 1:**
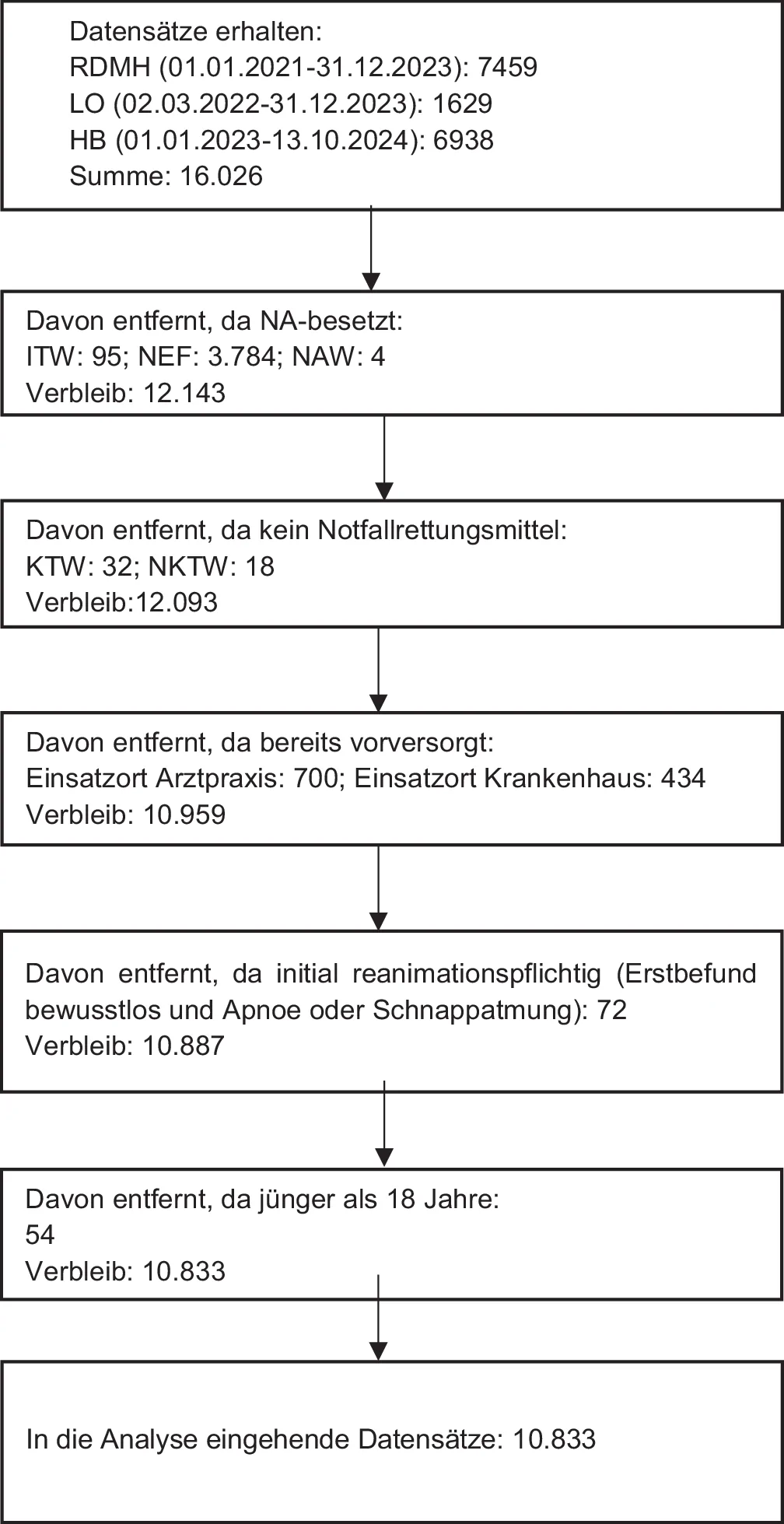
Datenzusammensetzung und -bereinigungsprozess

Die gesammelten Daten mussten umfangreich aneinander angepasst und bereinigt werden. Von dem RDMH und aus LO wurden je ein Protokoll pro Einsatzfahrzeug zur Verfügung gestellt, wobei in zusammenhängenden Einsätzen die gleichen Maßnahmen dokumentiert wurden. Diese inhaltsgleiche Dokumentation der bei einem Einsatz eingesetzten Teams wurde vor der Datenbereinigung stichprobenartig von 2 Autoren (TH und RK) an jeweils 50 Einsätzen überprüft und bestätigt. Die Daten aus HB wurden bereits zu Fällen zusammengeführt. Da NA-besetzte Rettungsmittel in der Regel nicht allein zum Einsatz kommen, wurden deren Protokolle aus der Analyse ausgeschlossen. Gleiches gilt für Protokolle von KTW oder NKTW, da hier davon auszugehen ist, dass mindestens ein RTW an der Versorgung beteiligt war und redundante Protokolldaten zu vermeiden waren. Weiterhin ausgeschlossen wurden Protokolle mit initial reanimationspflichtigen Patient:innen. Diese wurden anhand des Bewusstseinszustands „bewusstlos“ und der Atmung „Apnoe“ oder „Schnappatmung“ identifiziert. Hier ist davon auszugehen, dass die ergriffenen Maßnahmen primär der Reanimation und nicht der ACS-Versorgung dienen. Fehlende Werte im Bereich der Erstbefunde wurden mit „nicht dokumentiert“ erfasst. Abb. 1 gibt einen Überblick über Zusammensetzung, Zeiträume und Bereinigungsprozess der Daten. Das detaillierte Vorgehen zur Anpassung der Datenstrukturen sind dem Onlinesupplement zu entnehmen.

### Statistische Auswertung

Die so gewonnen Datensätze wurden anschließend durch einen Autor (TH) dichotom kodiert. Sind die zur Versorgung getroffenen Maßnahmen vollständig durch den Pyramidenprozess konsentiert durch NotSan abzuleisten, wurde diesen Patient:innen keine NA-Notwendigkeit beschieden. Wurde auch nur eine Maßnahme ergriffen, die nicht im Pyramidenprozess enthalten ist, wurde eine NA-Notwendigkeit kodiert.

Um die erste Forschungsfrage beantworten zu können, wurden die so gewonnenen Daten deskriptiv aufgearbeitet. Je nach Voraussetzungen erfolgt die Darstellung mit Mittelwerten (MW), Standardabweichungen (SD), Median (MD) und Interquartilsabstand (IQR). Die Normalverteilung wurde optisch mittels Q‑Q-Diagrammen geprüft. Bei nominalen Daten werden Häufigkeiten der gültigen Angaben berichtet.

Zur Beantwortung der zweiten Frage wurde mittels der dichotomen NA-Notwendigkeit eine logistische Regression durchgeführt. Die Erstbefunde wurden so umkodiert, dass diese möglicherweise im Notrufgespräch herausgearbeitet werden können. So wurde- beispielsweise der Erstbefund Atmung von ursprünglich 15 Ausprägungen auf 5 (unauffällig, Dyspnoe, Zyanose, Apnoe und „nicht dokumentiert“) umkodiert. Eingegangen sind dabei Prädiktoren, die möglicherweise bereits im Notrufgespräch abfragt oder erkennbar sind. Dies beinhaltete die umkodierten Erstbefunde von Bewusstsein, Atmung, Haut und Psyche und auch die Art des Einsatzorts (soweit *n* > 10), das Geschlecht, das Alter sowie die Schmerzskala. Als relevanten Einfluss wurden Chancenverhältnisse von mehr als 2 Punktwerten (Odds-Ratio [OR] von > 2) eingestuft. Die genaue Kodierung ist dem Onlinesupplement zu entnehmen.

Fehlende Daten wurden im Kodierungsprozess mit „nicht dokumentiert“ erfasst und flossen so in die Auswertung mit ein. Die statistischen Analysen wurden mittels JASP (Version 0.18.3; JASP-Team, Amsterdam, Niederlande) und Excel für Mac (Version: 16.91; Microsoft corp. Redmond, WA, USA) durchgeführt.

### Qualitätsaspekte

Die Berichterstattung erfolgt nach den „reporting guidelines“ STROBE für die Beobachtungsstudie und SAMPL für die Darstellung statistischer Ergebnisse [9, 18].

Für die Studie liegt ein positives Votum der Ethikkommission der HSD Hochschule Döpfer vom 31.05.2023 vor (Antragszeichen Beth_55_23) und sie wurde im Deutschen Register Klinischer Studien registriert (DRKS-ID: DRKS00034248). Die Daten sind in der angepassten und bereinigten Form über das Repositorium Zenodo abrufbar (http://www.https://doi.org/10.5281/zenodo.14192382).

## Ergebnisse

### Stichprobenbeschreibung

Insgesamt wurden 10.833 Datensätze aus den Protokollen extrahiert und gingen in die Analyse ein. Das Patient:innenkollektiv war im MW 65,7 Jahre alt (SD: 17,3) und eher männlich (57,6 %; *n* = 6244) als weiblich (41,8 %; *n* = 4531). Personen diversen Geschlechts wurden nicht dokumentiert; jedoch fehlen bei 58 (0,5 %) die Geschlechtsangabe, worin nonbinäre Personen enthalten sein könnten.

Die häufigsten Einsätze fanden im häuslichen Umfeld (82,9 %; *n* = 3226) und in Altenheimen (4,7 %; *n* = 128) statt. Jedoch fehlte in 6943 Protokollen eine Angabe des Einsatzorts. Tab. 1 bietet einen umfassenden Überblick über die Charakteristika der Stichprobe.

**Tab. 1:** Beschreibung der Stichprobe

Merkmal	Häufigkeit	Prozent*	Gültige Prozent*
*Gebiet*			
Bremen	6881	63,5	63,5
LK Oldenburg	638	5,9	5,9
Mittelhessen	3314	30,6	30,6
Nicht dokumentiert	0	0,0	–
*Art des Einsatzortes*			
Wohnung	3226	29,8	82,9
Altenheim	182	1,7	4,7
Arbeitsplatz	148	1,4	3,8
Bildungseinrichtung	1	0,0	0,0
Massenveranstaltung	3	0,0	0,1
Pflegeeinrichtung	12	0,1	0,3
Sonstige	111	1,0	2,9
Sportstätte	8	0,1	0,2
Straße	88	0,8	2,3
Öffentlicher Raum	111	1,0	2,9
Nicht dokumentiert	6943	64,1	–
*Geschlecht*			
Männlich	6244	57,6	58,0
Weiblich	4531	41,8	42,1
Nicht dokumentiert	58	0,5	–

*Auf eine Kommastelle gerundet

### Erstbefunde

Die Erstbefunde wurden ausgewertet, da diese den Patient:innenzustand zu einem möglichst frühen Zeitpunkt dokumentieren und damit möglicherweise bereits beim Notrufgespräch abgefragt werden können.

Der weit überwiegende Teil der Patient:innen war bei Bewusstsein (96,3 %; *n* = 10.173), hatte eine unauffällige Atmung (83,4 %, *n* = 8725), einen unauffälligen Hautzustand (81,6 %; *n* = 8643) und gab im MD 3 Punkte auf der Schmerzskala (IQR: 5) an. Das Onlinesupplement stellt die Erstbefunde detailliert deskriptiv dar.

### Durchgeführte Maßnahmen und verabreichte Medikamente

Die am häufigsten durchgeführte Maßnahme bei der ACS-Versorgung ist die Anlage eines Gefäßzugangs (32,5 %; *n* = 3518) gefolgt von der Sauerstoffgabe (5,9 %; *n* = 638). Insgesamt erhielten 35,2 % der Patient:innen ASS (*n* = 3818), gefolgt von Heparin (29,2 %; *n* = 3168). Dies sind alles Maßnahmen, die nach dem Pyramidenprozess den NotSan zuzuordnen sind. Die häufigsten NA-Interventionen waren die Gabe von Akrinor® (*n* = 82), die invasive Atemwegssicherung (*n* = 82), die Gabe von Magnesium (*n* = 49) und Noradrenalin (*n* = 48). Die erste Forschungsfrage beantwortend erhielten 2,5 % (*n* = 587) der Patient:innen eine Versorgung, die mit Bezug auf den Pyramidenprozess klar die Anwesenheit eines NA erfordert.

Das Onlinesupplement stellt die durchgeführten Maßnahmen und verabreichte Medikamente detailliert deskriptiv dar.

### Regressionsanalyse der Faktoren zu ärztlichen Maßnahmen

Das Modell der logistischen Regression ist als Ganzes signifikant (χ^2^ = 694,9; *p* < 0,001) und von mittlerer bis starker Modelgüte (Nagelkerke R^2^ 0,292; Effektstärke f^2^ 0,412, AUC 0,856; Vorhersagewahrscheinlichkeit: 97,7 %). Tab. 2 zeigt die Ergebnisse der Berechnungen.

**Tab. 2:** Logistische Regression zur NA-Notwendigkeit^a,b^

Koeffizienten				
	*n*	OR	95 %-Konfidenzintervall (OR)	*p*
*Alter*	–	1,008	0,998–1,018	0,105
*Geschlecht*	Referenz männlich (*n* = 6244)			
Weiblich	4531	0,994	0,744–1,327	0,966
*Bewusstsein*	Referenz wach (*n* = 10173)			
Nicht dokumentiert	268	2,726	1,357–5,475	0,005
Getrübt	341	7,771	5,308–11,376	< 0,001
Bewusstlos	51	24,501	11,416–52,584	< 0,001
*Atmung*	Referenz unauffällig (*n* = 8725)			
Nicht dokumentiert	371	9,067	5,651–14,547	< 0,001
Dyspnoe	1622	2,059	1,473–2,877	< 0,001
Zyanose	91	5,534	2,952–10,375	< 0,001
Apnoe	24	15,727	5,778–42,804	< 0,001
*Haut*	Referenz unauffällig (*n* = 8643)			
Nicht dokumentiert	236	1,241	0,579–2,660	0,580
Sonstige	906	1,404	0,906–2,175	0,129
Kaltschweißigkeit	1048	3,226	2,296–4,534	< 0,001
*Schmerz*	Referenz keiner (0; *n* = 1476)			
Nicht dokumentiert	5108	0,738	0,455–1,199	0,221
Leicht (1–3)	1634	0,537	0,341–0,847	0,008
Moderat (4–7)	2014	0,469	0,304–0,723	< 0,001
Stark (8–10)	601	0,461	0,259–0,819	0,008
*Einsatzort*	Referenz Wohnung (*n* = 3226)			
Nicht dokumentiert	6943	0,166	0,105–0,260	< 0,001
Arbeitsplatz	148	1,206	0,482–3,019	0,690
Pflegeeinrichtung	194	0,683	0,333–1,399	0,297
Öffentlicher Raum	211	0,666	0,306–1,449	0,305
Sonstiges	111	0,247	0,063–0,974	0,046
*Psyche*	Referenz unauffällig (*n* = 4407)			
Nicht dokumentiert	5312	1,486	1,047–2,110	0,027
Sonstiges	1065	1,161	0,749–1,799	0,506
Verwirrt	49	2,239	0,690–7,265	0,179

^a^NA-Notwendigkeit kodiert als Klasse 1.

^b^Gütekriterien: χ^2^ = 662,665; *p* < 0,001, Nagelkerke R^2^ = 0,292, Effektstärke f^2^ = 0,412

Die als Prädiktoren eingehenden Erstbefunde werden, aufgeteilt nach der NA-Notwendigkeit, im Onlinesupplement detailliert deskriptiv dargestellt.

Signifikant und mit einer OR größer 2 wirken ein nicht dokumentiertes (*p* = 0,005; OR 2,726; 95-KI 1,357–5,475), getrübtes (*p* < 0,001; OR 7,771; 95-KI 5,308–11,376) oder nicht vorhandenes Bewusstsein (*p* < 0,001; OR 24,501; 95-KI 11,416–52,584), eine nicht dokumentierte Atmung (*p* < 0,001; OR 9,067; 95-KI 5,651–14,547), eine Dyspnoe (*p* < 0,001; OR 2,059; 95-KI 1,473–2,877), eine Zyanose (*p* < 0,001; OR 5,534; 95-KI 2,952–10,375), eine Apnoe (*p* < 0,001; OR 15,727; 95-KI 5,778–42,804) sowie die Kaltschweißigkeit (*p* < 0,001; OR 3,226; 95-KI 2,296–4,534) ein.

Patient:innen, die initial eine Einschränkung des Bewusstseins, eine Beeinträchtigung der Atmung oder eine Kaltschweißigkeit aufweisen, haben eine mindestens doppelt so hohe Wahrscheinlichkeit, eine NA-Maßnahme zu erhalten, als Patient:innen mit jeweiligen Normalbefunden. Dies ist die Antwort auf die zweite Forschungsfrage.

## Diskussion

### Einordnung und Interpretation der Ergebnisse

Patient:innen, die durch den Rettungsdienst mit einer (Verdachts‑)Diagnose ACS versorgt werden, erhalten in 2,5 % – und damit ziemlich genau in jedem 40. Fall – eine Intervention, die, basierend auf dem Pyramidenprozess, nicht durch eine NotSan hätte durchgeführt werden können. Dies reiht sich in einige internationale Erkenntnisse ein, die aufzeigen, dass gefährliche Events im Rahmen der rettungsdienstlichen ACS-Versorgung eher selten zu erwarten sind [6, 23].

Ein eingeschränktes oder fehlendes Bewusstsein erhöht maßgeblich die Wahrscheinlichkeit, dass NA-Maßnahmen ergriffen wurden. Somit reiht sich dieser Ergebnisaspekt ein in eine Reihe internationaler Untersuchungen, die reduziertes Bewusstsein in verschiedenen Kontexten als Prädiktor für schwere Verläufe herausgearbeitet haben [4, 11, 21].

Beim Erstbefund der Atmung ließen alle pathologischen Befunde einen Schluss auf eine deutliche Erhöhung der Inzidenz von NA-Maßnahmen zu. Die Dyspnoe überstieg nur knapp die Berichtsgrenze von OR > 2. Dies mag daran liegen, dass die Ursachen für Probleme mit der Atmung mannigfaltig und nicht immer auf schwerwiegende Störungen zurückzuführen sind [12]. Die Zyanose hingegen ist häufig ein Zeichen für Hypoxämie, die – zumindest akut eingetreten – unmittelbar behandlungsbedürftig ist [22]. Dasselbe gilt auch für die Apnoe.

Kaltschweißigkeit als weiterer Zustand, der die Wahrscheinlichkeit einer NA-Maßnahme deutlich erhöht, ist schon lange als Prädiktor für schwere Verläufe im Bereich der kardiologischen Notfallmedizin bekannt [13, 25].

### Limitierungen

Die Studie geht mit einer Reihe an Limitierungen einher. Zunächst basiert sie auf der Analyse von Rettungsdienst- und NA-Protokollen, damit sind die Ergebnisse stark abhängig von der Dokumentationsqualität.

Als Einschlusskriterien wurden die (Verdachts‑)Diagnosen der Protokolle herangezogen. Dies kann einen Selection-Bias verursachen, da nicht bekannt ist, ob diese Patient:innen durch die Notrufabfrage bereits entsprechend identifiziert wurden, oder aber Patient:innen, die durch die Leitstelle als ACS verschlagwortet wurden, durch das Team vor Ort eine ganz andere (Verdachts‑)Diagnose erhielten. Weiterhin war es bei den Protokollen aus RDMH und LO möglich, mehrere Diagnosen zu dokumentieren. Somit wurden mutmaßlich auch Datensätze eingeschlossen, bei denen der ACS-Verdacht eher eine Ausschlussdiagnose war. Dies könnte auch eine Erklärung für die überschaubare Maßnahmeninzidenz sein.

Die Daten stammen aus unterschiedlichen Rettungsdienstorganisationen mit variierenden digitalen Erfassungssystemen und Zeiträumen, was die Vergleichbarkeit der Datensätze beeinträchtigen könnte. Durch Ausschluss von Datensätzen, wie z. B. von reanimationspflichtigen Patient:innen, könnten wichtige Informationen über spezifische Patient:innengruppen verloren gegangen sein.

Die nicht dokumentierten Erstbefunde bei Bewusstsein und Atmung waren ebenfalls mit einer deutlich erhöhten NA-Notwendigkeit verbunden. Welche Zustände sich hinter dieser Kategorie befinden, lässt sich nicht erschließen. Hier sind weitere Studien zur Untersuchung der Dokumentationsqualität im Rettungsdienst aber auch zur Identifizierung möglicher weiterer Prädiktoren notwendig.

Basierend auf dem Pyramidenprozess wurden Maßnahmen als nicht NA-Intervention kategorisiert, die auf eine kardiozirkulatorische Instabilität hinweisen könnten (z. B. Amiodaron, Adrenalin, Kardioversion). Dennoch sollte dieser Aspekt bei der Interpretation der Ergebnisse berücksichtigt werden, da er die Aussage zur geringen Inzidenz notärztlicher Maßnahmen potenziell leicht relativieren kann. Ob und unter welchen Voraussetzungen diese Patient:innengruppe von einer primären NA-Alarmierung profitiert oder ob eine Nachforderung Auswirkungen auf Output und Outcome hat, sollte in weiteren Studien adressiert werden.

### Generalisierbarkeit

Die hier vorliegende retrospektive Studie arbeitet explorativ die Notwendigkeit des NA-Einsatzes im Kontext der rettungsdienstlichen ACS-Versorgung auf. Diese Frage ist so komplex, dass sie nicht mit einer einzigen Beobachtungsstudie abschließend beantwortet werden kann. Unter Berücksichtigung der aufgeführten Limitationen, aber auch der großen Fallzahl kann man davon ausgehen, dass die Inzidenz der NA-Maßnahmen auf Basis des Pyramidenprozesses bei dieser Patient:innenklientel relativ gering ist [1, 2].

Abschließend soll erneut darauf hingewiesen werden, dass diese Studie sich nur auf die Inzidenz und Vorhersage notärztlicher Maßnahmen bezieht. Es gibt aber bereits Indizien, dass beispielweise die Leitlinienadhärenz nicht durch die Anwesenheit eines/einer NA erhöht wird. Dennoch geht der ärztliche Beitrag in der ACS-Versorgung über die reine Maßnahmendurchführung hinaus. Entsprechende Kompetenz könnte zukünftig durch die optionale Beratung durch eine/einen Telenotärzt:in, die bessere bzw. andere Ausbildung von NotSan oder die Einbindung von Künstlicher Intelligenz an die Einsatzstelle gebracht werden [16, 24]. So gibt es beispielsweise Hinweise darauf, dass Künstliche Intelligenz zukünftig die – verbesserungswürdigen – EKG-Interpretationsfähigkeiten übernehmen kann [5, 10].

## Fazit für die Praxis

- Auf Basis dieser Studie erscheint es mit Perspektive auf die Maßnahmen nicht nötig, dass jeder/jede ACS-Patient:in durch einen NA versorgt werden muss. Patient:innen, die im Rahmen des Notrufgesprächs ein reduziertes Bewusstsein oder eine Zyanose erkennen lassen, sollten jedoch auch weiterhin primär gemeinsam von NotSan und NA versorgt werden.
- Jedoch sind noch einige Fragen und Perspektiven durch – insbesondere – prospektive Studien zu adressieren, denn der Fokus allein auf die Maßnahmen reduziert die ärztliche Tätigkeit im Kontext der rettungsdienstlichen Versorgung möglichweise unzulässig.

## Supplementary Information

## Data Availability

Die erhobenen Datensätze sind verfügbar im Repositorium Zenodo unter URL: http://www.https://doi.org/10.5281/zenodo.14192382
