# Supplementary material for: Emergency physician interventions in the care of patients with acute coronary syndrome: Comparative, retrospective, observational study
Source: Med Klin Intensivmed Notfmed. 2025 Aug 1;121(6):465–72. [Article in German] doi: 10.1007/s00063-025-01306-4 (PMC13538223; doi:10.1007/s00063-025-01306-4)
Supplement: Supplementary file 1 [file 63_2025_1306_MOESM1_ESM.docx]

Onlinesupplement zu

**Notärztliche Maßnahmen bei der Versorgung von Patient:innen mit Akutem Coronarsyndrom**

Eine vergleichende, retrospektive Beobachtungsstudie

Thomas Hofmann^1 2 3^, Lena Himmelreich^2^, Roland Kirschenlohr^3^, Toni Fredrich^2 3 4^, Patrick Andreas Eder^3^ ^8^, Frank Flake^6^, Kathrin Bagdahn^4^, Jan Orendt^7^, Melanie Reuter-Oppermann^3 9^, Rolf Lefering^1^

DOI: https://doi.org/10.1007/s00063-025-01306-4

^1^ Institut für Forschung in der Operativen Medizin (IFOM), Fakultät für Gesundheit, Universität Witten/Herdecke, Köln, , Deutschland

^2^ HSD Hochschule Döpfer University of Applied Sciences, Fachbereich Gesundheit und Soziales, Potsdam, Deutschland

^3^ Deutsche Gesellschaft für Rettungswissenschaften (DGRe), Aachen, Deutschland

^4^ Feuerwehr Bremen, Bremen, Deutschland

^6^ Landkreis Oldenburg, Wildeshausen, Deutschland

^7^ DRK Rettungsdienst Mittelhessen, Marburg, Deutschland

^8^ ZTM, Innovationsmanagement, Bad Kissingen, Deutschland

^9^ ILS Mannheim gGmbH, Mannheim, Deutschland

**Inhaltsverzeichnis**

[Zusammenführung der Daten	3](#_Toc196291778)

[Codierung der Daten	5](#_Toc196291779)

[Zusammenfassung einiger Medikamente	5](#_Toc196291780)

[Zusammenfassung der Erstbefunde	5](#_Toc196291781)

[Umkodierung EB-Atmung	5](#_Toc196291782)

[Umkodierung EB-Bewusstsein	6](#_Toc196291783)

[Umkodierung EB-Haut	6](#_Toc196291784)

[Umkodierung EB-Schmerz	6](#_Toc196291785)

[Umkodierung EB-Psyche	7](#_Toc196291786)

[Deskription der Erstbefunde	8](#_Toc196291787)

[Deskription ohne Zusammenführung für die logistische Regression	8](#_Toc196291788)

[Häufigkeiten für EB - Neurologie - Bewusstsein	8](#_Toc196291789)

[Häufigkeiten für EB - Atmung	9](#_Toc196291790)

[Häufigkeiten für EB - Untersuchung - Haut	9](#_Toc196291791)

[Häufigkeiten für EB - Untersuchung - Schmerzen	10](#_Toc196291792)

[Häufigkeiten für EB - Psyche	10](#_Toc196291793)

[Deskription nach Zusammenführung der Erstbefunde und der NA-Notwendigkeit	11](#_Toc196291794)

[Häufigkeiten für NA-Notwendigkeit nach EB-Bewusstsein	11](#_Toc196291795)

[Häufigkeiten für NA-Notwendigkeit nach EB-Atmung	12](#_Toc196291796)

[Häufigkeiten für NA-Notwendigkeit nach EB-Haut	13](#_Toc196291797)

[Häufigkeiten für NA-Notwendigkeit nach EB-Schmerz	14](#_Toc196291798)

[Häufigkeiten für NA-Notwendigkeit nach EB Psyche	15](#_Toc196291799)

[Häufigkeiten für NA-Notwendigkeit nach Einsatzort	16](#_Toc196291800)

[Deskription der Maßnahmen und verabreichten Medikamente	17](#_Toc196291801)

[Deskription der Maßnahmen	17](#_Toc196291802)

[Häufigkeiten für Atemwege freimachen	17](#_Toc196291803)

[Häufigkeiten für Entlastungspunktion	17](#_Toc196291804)

[Häufigkeiten für invasive Atemwegssicherung	17](#_Toc196291805)

[Häufigkeiten für Sauerstoffgabe	17](#_Toc196291806)

[Häufigkeiten für Defibrillation	17](#_Toc196291807)

[Häufigkeiten für Gefäßzugang	17](#_Toc196291808)

[Häufigkeiten für Kardioversion	18](#_Toc196291809)

[Häufigkeiten für Pacer/Schrittmacher	18](#_Toc196291810)

[Deskription der Medikamente	19](#_Toc196291811)

[Zuordnung von Medikamenten und Maßnahmen zum Pyramidenprozess	21](#_Toc196291812)

# Zusammenführung der Daten

Datenstruktur von OL und MH waren gleich und wurden mit den Daten aus HB zusammengeführt.

| Item (Spalte) | Variable LO/MH | Variable HB | Variable Zusammenführung |
| --- | --- | --- | --- |
| Geschlecht (G) | W | Weiblich | Weiblich |
| Geschlecht (G) | M | Männlich | Männlich |
| Geschlecht (G) | Leer | Unbekannt | Leer |
| Geschlecht (G) | NULL |  | Leer |
| Geschlecht (G) | Unbekannt |  | Leer |
| EB-Neurologie-Bewusstsein^1^ (I) | Wach | Wach/orientiert | Wach |
| EB-Neurologie-Bewusstsein^1^ (I) | Reagiert auf Ansprache | Reaktion auf Ansprache | Reaktion auf Ansprache |
| EB-Neurologie-Bewusstsein^1^ (I) | Reagiert auf Schmerzreiz | Reaktion auf Schmerzreiz | Reaktion auf Schmerzreiz |
| EB-Neurologie-Bewusstsein^1^ (I) |  | Getrübt | Reaktion auf Ansprache |
| EB-Neurologie-Bewusstsein^1^ (I) | NULL |  | Nicht dokumentiert |
| EB-Neurologie-Bewusstsein^1^ (I) | Sonstige |  | Nicht dokumentiert |
| EB-Neurologie-Bewusstsein^1^ (I) | Nicht untersucht |  | Nicht dokumentiert |
| EB-Neurologie-Bewusstsein^1^ (I) | TRUE |  | Nicht dokumentiert |
| EB-Neurologie-Bewusstsein^1^ (I) | Wach / ansprechbar |  | Wach |
| EB-Atmung^1^ (R) | Unauffällig / Spontanatmung | Unauffällig | Unauffällig |
| EB-Atmung^1^ (R) | Belastungsdyspnoe |  | Dyspnoe |
| EB-Atmung^1^ (R) | Ruhedyspnoe |  | Dyspnoe |
| EB-Atmung^1^ (R) | Bradypnoe |  | Dyspnoe |
| EB-Atmung^1^ (R) | Tachypnoe |  | Dyspnoe |
| EB-Atmung^1^ (R) | FALSE |  | Leer |
| EB-Atmung^1^ (R) | NULL |  | Leer |
| EB-Atmung^1^ (R) | Nicht beurteilbar |  | Leer |
| EB-Atmung^1^ (R) | Beatmung |  | Apnoe |
| EB-Atmung^1^ (R) | Atemwegsverlegung |  | Apnoe |
| EB-Atmung^1^ (R) |  | Atmung nicht untersucht | Nicht dokumentiert |
| EB-Haut^1^ (AD) | FALSE |  | Nicht dokumentiert |
| EB-Haut^1^ (AD) | NULL |  | Nicht dokumentiert |
| EB-Haut^1^ (AD) | Nicht untersucht |  | Nicht dokumentiert |
| EB-Haut^1^ (AD) |  | Haut – unauffällig | Unauffällig |
| EB-Haut^1^ (AD) |  | Nicht untersucht | Leer |
| EB-Haut^1^ (AD) | Kaltschweißigkeit | Kaltschweißig | Kaltschweißigkeit |
| EB-Haut^1^ (AD) | Sonstige | pathologisch | Sonstige |
| EB-Psyche^1^ (DW) | Unauffällig | Psyche – unauffällig | Unauffällig |
| EB-Psyche^1^ (DW) | Motorisch unruhig | Unruhig | Motorisch unruhig |
| EB-Psyche^1^ (DW) | Nicht untersucht |  | Nicht dokumentiert |
| EB-Psyche^1^ (DW) | NULL |  | Nicht dokumentiert |
| EB-Untersuchung Schmerz (EJ) | NULL |  | Leer |
| EB-Untersuchung Schmerz (EJ) | Nicht beurteilbar |  | Leer |
| AZ vor Ereignis (EK) |  |  |  |

Anmerkungen:

Anpassungen bei offensichtlichen Tipp-, Übertragungsfehler (Umlaute) oder einfache Unterschiede in der Syntax (z.B. Leerzeichen) wurden nicht in der Tabelle erfasst

^1^: Bei einigen Items waren Mehrfachnennungen möglich (z.B. wach & Reaktion auf Ansprache). In diesen Fällen wurde jeweils auf die Variable reduziert, die eine bzw. die stärkere Beeinträchtigung des jeweiligen Items darstellt.

# Codierung der Daten

## Zusammenfassung einiger Medikamente

| Antiemetikum | Dimenhydrnat  Ondansetron  Metocopramid |
| --- | --- |
| Opiat | Piritramin  Morphin  Fentanyl  Sufentanil |
| Hypnotikum | Etomidate  Propofol |
| Muskelrelaxans | Pancuronium  Rocuronium  Succinylcholin |
| Katecholamine – ohne Adrenalin | Dobutamin  Dopamin  Noradrenalin |

## Zusammenfassung der Erstbefunde

Für die logistische Regression wurden die Erstbefunde so zusammengefasst, dass diese über das Telefon im Rahmen des Notrufgesprächs erfragbar erscheinen.

| Umkodierung EB-Atmung | |
| --- | --- |
| **Ursprünglicher Code** | **Neuer Code (EBr2-Atmung)** |
| unauffällig | unauffällig |
| Dyspnoe | Dyspnoe |
| Hyperventilation |  |
| Rasselgeräusche |  |
| Ruhedyspnoe |  |
| Schnappatmung |  |
| Spastik |  |
| Stridor |  |
| Tachypnoe |  |
| sonstige path. Atemmuster |  |
| Zyanose | Zyanose |
| Apnoe | Apnoe |
| Schnappatmung |  |
| Beatmung |  |
| Nicht dokumentiert | Nicht dokumentiert |

| Umkodierung EB-Bewusstsein | |
| --- | --- |
| **Ursprünglicher Code** | **Neuer Code (EBr2-Bewusstsein)** |
| Wach | unauffällig |
| Reaktion auf Ansprache | getrübt |
| Reaktion auf Schmerzreiz |  |
| Bewusstlos | bewusstlos |
| Nicht dokumentiert | Nicht dokumentiert |
|  |  |

| Umkodierung EB-Haut | |
| --- | --- |
| **Ursprünglicher Code** | **Neuer Code (EBr2-Haut)** |
| unauffällig | unauffällig |
| Dekubitus | Sonstige |
| Exanthem |  |
| Sonstige |  |
| Stehende Hautfalten |  |
| Ödeme |  |
| Kaltschweißigkeit | Kaltschweißigkeit |
| Nicht dokumentiert | Nicht dokumentiert |
|  |  |

| Umkodierung EB-Schmerz | |
| --- | --- |
| **Ursprünglicher Code** | **Neuer Code (EBr2-Schmerz)** |
| 0 | Keiner (0) |
| 1 | Leicht (1-3) |
| 2 |  |
| 3 |  |
| 4 | Moderat (4-7) |
| 5 |  |
| 6 |  |
| 7 |  |
| 8 | Stark (8-10) |
| 9 |  |
| 10 |  |
| Nicht dokumentiert | Nicht dokumentiert |
|  |  |

| Umkodierung EB-Psyche | |
| --- | --- |
| **Ursprünglicher Code** | **Neuer Code (EBr2-Psyche)** |
| unauffällig | unauffällig |
| Sonstige | Sonstige |
| Aggressiv |  |
| Depressiv |  |
| Erregt |  |
| Euphorisch |  |
| Motorisch unruhig |  |
| Suizidal |  |
| Verlangsamt |  |
| Wahnhaft |  |
| ängstlich |  |
| Verwirrt | Verwirrt |
| Nicht dokumentiert | Nicht dokumentiert |
|  |  |

| **Umkodierung Einsatzort für Log. Regression** | |
| --- | --- |
| **Ursprünglicher Code** | **Neuer Code (EBr2-Schmerz)** |
| Wohnung | Wohnung |
| Altenheim | Pflegeeinrichtung |
| Pflegeeinrichtung |  |
| Bildungseinrichtung | Öffentlicher Raum |
| Massenveranstaltung |  |
| Sportstätte |  |
| Straße |  |
| Öffentlicher Raum |  |
| Arbeitsplatz | Arbeitsplatz |
| Sonstiges | Sonstiges |
| Nicht dokumentiert | Nicht dokumentiert |
|  |  |

# Deskription der Erstbefunde

## Deskription ohne Zusammenführung für die logistische Regression

| Häufigkeiten für EB - Neurologie - Bewusstsein | | | | | | | | | |
| --- | --- | --- | --- | --- | --- | --- | --- | --- | --- |
| EB - Neurologie - Bewusstsein | | Häufigkeit | | Prozent | | Prozent gültig | | Prozent kumulativ | |
| wach |  | 10173 |  | 93.908 |  | 96.290 |  | 96.290 |  |
| Reaktion auf Ansprache |  | 255 |  | 2.354 |  | 2.414 |  | 98.703 |  |
| Reaktion auf Schmerzreiz |  | 31 |  | 0.286 |  | 0.293 |  | 98.997 |  |
| analgosediert/Narkose |  | 55 |  | 0.508 |  | 0.521 |  | 99.517 |  |
| bewusstlos |  | 51 |  | 0.471 |  | 0.483 |  | 100.000 |  |
| Nicht dokumentiert |  | 268 |  | 2.474 |  |  |  |  |  |
| Gesamt |  | 10833 |  | 100.000 |  |  |  |  |  |
|  |  |  |  |  |  |  |  |  |  |

| Häufigkeiten für EB - Atmung | | | | | | | | | |
| --- | --- | --- | --- | --- | --- | --- | --- | --- | --- |
| EB - Atmung | | Häufigkeit | | Prozent | | Prozent gültig | | Prozent kumulativ | |
| unauffällig |  | 8725 |  | 80.541 |  | 83.397 |  | 83.397 |  |
| Apnoe |  | 11 |  | 0.102 |  | 0.105 |  | 83.502 |  |
| Beatmung |  | 3 |  | 0.028 |  | 0.029 |  | 83.531 |  |
| Dyspnoe |  | 1209 |  | 11.160 |  | 11.556 |  | 95.087 |  |
| Hyperventilation |  | 155 |  | 1.431 |  | 1.482 |  | 96.569 |  |
| Rasselgeräusche |  | 102 |  | 0.942 |  | 0.975 |  | 97.543 |  |
| Ruhedyspnoe |  | 17 |  | 0.157 |  | 0.162 |  | 97.706 |  |
| Schnappatmung |  | 10 |  | 0.092 |  | 0.096 |  | 97.802 |  |
| Spastik |  | 92 |  | 0.849 |  | 0.879 |  | 98.681 |  |
| Stridor |  | 18 |  | 0.166 |  | 0.172 |  | 98.853 |  |
| Tachypnoe |  | 12 |  | 0.111 |  | 0.115 |  | 98.968 |  |
| Zyanose |  | 91 |  | 0.840 |  | 0.870 |  | 99.838 |  |
| sonstige path. Atemmuster |  | 17 |  | 0.157 |  | 0.162 |  | 100.000 |  |
| Nicht dokumentiert |  | 371 |  | 3.425 |  |  |  |  |  |
| Gesamt |  | 10833 |  | 100.000 |  |  |  |  |  |
|  | | | | | | | | | |

| Häufigkeiten für EB - Untersuchung - Haut | | | | | | | | | |
| --- | --- | --- | --- | --- | --- | --- | --- | --- | --- |
| EB - Untersuchung - Haut | | Häufigkeit | | Prozent | | Prozent gültig | | Prozent kumulativ | |
| unauffällig |  | 8643 |  | 79.784 |  | 81.561 |  | 81.561 |  |
| Dekubitus |  | 1 |  | 0.009 |  | 0.009 |  | 81.570 |  |
| Exanthem |  | 9 |  | 0.083 |  | 0.085 |  | 81.655 |  |
| Kaltschweißigkeit |  | 1048 |  | 9.674 |  | 9.890 |  | 91.545 |  |
| Sonstige |  | 91 |  | 0.840 |  | 0.859 |  | 92.404 |  |
| stehende Hautfalten |  | 638 |  | 5.889 |  | 6.021 |  | 98.424 |  |
| Ödeme |  | 167 |  | 1.542 |  | 1.576 |  | 100.000 |  |
| Nicht dokumentiert |  | 236 |  | 2.179 |  |  |  |  |  |
| Gesamt |  | 10833 |  | 100.000 |  |  |  |  |  |
|  |  |  |  |  |  |  |  |  |  |

| Häufigkeiten für EB - Untersuchung - Schmerzen | | | | | | | | | |
| --- | --- | --- | --- | --- | --- | --- | --- | --- | --- |
| EB - Untersuchung - Schmerzen | | Häufigkeit | | Prozent | | Prozent gültig | | Prozent kumulativ | |
| 0 |  | 1476 |  | 13.625 |  | 25.782 |  | 25.782 |  |
| 1 |  | 283 |  | 2.612 |  | 4.943 |  | 30.725 |  |
| 2 |  | 599 |  | 5.529 |  | 10.463 |  | 41.188 |  |
| 3 |  | 752 |  | 6.942 |  | 13.135 |  | 54.323 |  |
| 4 |  | 558 |  | 5.151 |  | 9.747 |  | 64.070 |  |
| 5 |  | 702 |  | 6.480 |  | 12.262 |  | 76.332 |  |
| 6 |  | 396 |  | 3.655 |  | 6.917 |  | 83.249 |  |
| 7 |  | 358 |  | 3.305 |  | 6.253 |  | 89.502 |  |
| 8 |  | 393 |  | 3.628 |  | 6.865 |  | 96.367 |  |
| 9 |  | 106 |  | 0.978 |  | 1.852 |  | 98.218 |  |
| 10 |  | 102 |  | 0.942 |  | 1.782 |  | 100.000 |  |
| Nicht dokumentiert |  | 5108 |  | 47.152 |  |  |  |  |  |
| Gesamt |  | 10833 |  | 100.000 |  |  |  |  |  |
|  | | | | | | | | | |

| Häufigkeiten für EB - Psyche | | | | | | | | | |
| --- | --- | --- | --- | --- | --- | --- | --- | --- | --- |
| EB-Psyche | | Häufigkeit | | Prozent | | Prozent gültig | | Prozent kumulativ | |
| Sonstige |  | 3 |  | 0.028 |  | 0.054 |  | 0.054 |  |
| aggressiv |  | 9 |  | 0.083 |  | 0.163 |  | 0.217 |  |
| depressiv |  | 46 |  | 0.425 |  | 0.833 |  | 1.051 |  |
| erregt |  | 256 |  | 2.363 |  | 4.637 |  | 5.687 |  |
| euphorisch |  | 7 |  | 0.065 |  | 0.127 |  | 5.814 |  |
| motorisch unruhig |  | 54 |  | 0.498 |  | 0.978 |  | 6.792 |  |
| sonstige |  | 12 |  | 0.111 |  | 0.217 |  | 7.010 |  |
| suizidal |  | 2 |  | 0.018 |  | 0.036 |  | 7.046 |  |
| unauffällig |  | 4407 |  | 40.681 |  | 79.822 |  | 86.868 |  |
| verlangsamt |  | 162 |  | 1.495 |  | 2.934 |  | 89.803 |  |
| verwirrt |  | 49 |  | 0.452 |  | 0.888 |  | 90.690 |  |
| wahnhaft |  | 5 |  | 0.046 |  | 0.091 |  | 90.781 |  |
| ängstlich |  | 509 |  | 4.699 |  | 9.219 |  | 100.000 |  |
| Nicht dokumentiert |  | 5312 |  | 49.035 |  |  |  |  |  |
| Gesamt |  | 10833 |  | 100.000 |  |  |  |  |  |
|  | | | | | | | | | |

## Deskription nach Zusammenführung der Erstbefunde und der NA-Notwendigkeit

| Häufigkeiten für NA-Notwendigkeit nach EB-Bewusstsein | | | | | | | | | | | |
| --- | --- | --- | --- | --- | --- | --- | --- | --- | --- | --- | --- |
| EBr2-Bewusstsein | | NA-Notwendigkeit | | Häufigkeit | | Prozent | | Prozent gültig | | Prozent kumulativ | |
| wach |  | Nein |  | 10011 |  | 98.408 |  | 98.408 |  | 98.408 |  |
|  |  | Ja |  | 162 |  | 1.592 |  | 1.592 |  | 100.000 |  |
|  |  | Fehlend |  | 0 |  | 0.000 |  |  |  |  |  |
|  |  | Gesamt |  | 10173 |  | 100.000 |  |  |  |  |  |
| nicht dokumentiert |  | Nein |  | 255 |  | 95.149 |  | 95.149 |  | 95.149 |  |
|  |  | Ja |  | 13 |  | 4.851 |  | 4.851 |  | 100.000 |  |
|  |  | Fehlend |  | 0 |  | 0.000 |  |  |  |  |  |
|  |  | Gesamt |  | 268 |  | 100.000 |  |  |  |  |  |
| getrübt |  | Nein |  | 280 |  | 82.111 |  | 82.111 |  | 82.111 |  |
|  |  | Ja |  | 61 |  | 17.889 |  | 17.889 |  | 100.000 |  |
|  |  | Fehlend |  | 0 |  | 0.000 |  |  |  |  |  |
|  |  | Gesamt |  | 341 |  | 100.000 |  |  |  |  |  |
| bewusstlos |  | Nein |  | 21 |  | 41.176 |  | 41.176 |  | 41.176 |  |
|  |  | Ja |  | 30 |  | 58.824 |  | 58.824 |  | 100.000 |  |
|  |  | Fehlend |  | 0 |  | 0.000 |  |  |  |  |  |
|  |  | Gesamt |  | 51 |  | 100.000 |  |  |  |  |  |
|  | | | | | | | | | | | |

| Häufigkeiten für NA-Notwendigkeit nach EB-Atmung | | | | | | | | | | | |
| --- | --- | --- | --- | --- | --- | --- | --- | --- | --- | --- | --- |
| EBr2-Atmung | | NA-Notwendigkeit | | Häufigkeit | | Prozent | | Prozent gültig | | Prozent kumulativ | |
| unauffällig |  | Nein |  | 8612 |  | 98.705 |  | 98.705 |  | 98.705 |  |
|  |  | Ja |  | 113 |  | 1.295 |  | 1.295 |  | 100.000 |  |
|  |  | Fehlend |  | 0 |  | 0.000 |  |  |  |  |  |
|  |  | Gesamt |  | 8725 |  | 100.000 |  |  |  |  |  |
| nicht dokumentiert |  | Nein |  | 331 |  | 89.218 |  | 89.218 |  | 89.218 |  |
|  |  | Ja |  | 40 |  | 10.782 |  | 10.782 |  | 100.000 |  |
|  |  | Fehlend |  | 0 |  | 0.000 |  |  |  |  |  |
|  |  | Gesamt |  | 371 |  | 100.000 |  |  |  |  |  |
| Dyspnoe |  | Nein |  | 1551 |  | 95.623 |  | 95.623 |  | 95.623 |  |
|  |  | Ja |  | 71 |  | 4.377 |  | 4.377 |  | 100.000 |  |
|  |  | Fehlend |  | 0 |  | 0.000 |  |  |  |  |  |
|  |  | Gesamt |  | 1622 |  | 100.000 |  |  |  |  |  |
| Zyanose |  | Nein |  | 61 |  | 67.033 |  | 67.033 |  | 67.033 |  |
|  |  | Ja |  | 30 |  | 32.967 |  | 32.967 |  | 100.000 |  |
|  |  | Fehlend |  | 0 |  | 0.000 |  |  |  |  |  |
|  |  | Gesamt |  | 91 |  | 100.000 |  |  |  |  |  |
| Apnoe |  | Nein |  | 12 |  | 50.000 |  | 50.000 |  | 50.000 |  |
|  |  | Ja |  | 12 |  | 50.000 |  | 50.000 |  | 100.000 |  |
|  |  | Fehlend |  | 0 |  | 0.000 |  |  |  |  |  |
|  |  | Gesamt |  | 24 |  | 100.000 |  |  |  |  |  |
|  | | | | | | | | | | | |

| Häufigkeiten für NA-Notwendigkeit nach EB-Haut | | | | | | | | | | | |
| --- | --- | --- | --- | --- | --- | --- | --- | --- | --- | --- | --- |
| EBr2-Haut | | NA-Notwendigkeit | | Häufigkeit | | Prozent | | Prozent gültig | | Prozent kumulativ | |
| unauffällig |  | Nein |  | 8516 |  | 98.531 |  | 98.531 |  | 98.531 |  |
|  |  | Ja |  | 127 |  | 1.469 |  | 1.469 |  | 100.000 |  |
|  |  | Fehlend |  | 0 |  | 0.000 |  |  |  |  |  |
|  |  | Gesamt |  | 8643 |  | 100.000 |  |  |  |  |  |
| nicht dokumentiert |  | Nein |  | 224 |  | 94.915 |  | 94.915 |  | 94.915 |  |
|  |  | Ja |  | 12 |  | 5.085 |  | 5.085 |  | 100.000 |  |
|  |  | Fehlend |  | 0 |  | 0.000 |  |  |  |  |  |
|  |  | Gesamt |  | 236 |  | 100.000 |  |  |  |  |  |
| Sonstige |  | Nein |  | 862 |  | 95.143 |  | 95.143 |  | 95.143 |  |
|  |  | Ja |  | 44 |  | 4.857 |  | 4.857 |  | 100.000 |  |
|  |  | Fehlend |  | 0 |  | 0.000 |  |  |  |  |  |
|  |  | Gesamt |  | 906 |  | 100.000 |  |  |  |  |  |
| Kaltschweißigkeit |  | Nein |  | 965 |  | 92.080 |  | 92.080 |  | 92.080 |  |
|  |  | Ja |  | 83 |  | 7.920 |  | 7.920 |  | 100.000 |  |
|  |  | Fehlend |  | 0 |  | 0.000 |  |  |  |  |  |
|  |  | Gesamt |  | 1048 |  | 100.000 |  |  |  |  |  |
|  | | | | | | | | | | | |

| Häufigkeiten für NA-Notwendigkeit nach EB-Schmerz | | | | | | | | | | | |
| --- | --- | --- | --- | --- | --- | --- | --- | --- | --- | --- | --- |
| EBr2-Schmerz | | NA-Notwendigkeit | | Häufigkeit | | Prozent | | Prozent gültig | | Prozent kumulativ | |
| Keiner (0) |  | Nein |  | 1406 |  | 95.257 |  | 95.257 |  | 95.257 |  |
|  |  | Ja |  | 70 |  | 4.743 |  | 4.743 |  | 100.000 |  |
|  |  | Fehlend |  | 0 |  | 0.000 |  |  |  |  |  |
|  |  | Gesamt |  | 1476 |  | 100.000 |  |  |  |  |  |
| nicht dokumentiert |  | Nein |  | 5007 |  | 98.023 |  | 98.023 |  | 98.023 |  |
|  |  | Ja |  | 101 |  | 1.977 |  | 1.977 |  | 100.000 |  |
|  |  | Fehlend |  | 0 |  | 0.000 |  |  |  |  |  |
|  |  | Gesamt |  | 5108 |  | 100.000 |  |  |  |  |  |
| Leicht (1-3) |  | Nein |  | 1601 |  | 97.980 |  | 97.980 |  | 97.980 |  |
|  |  | Ja |  | 33 |  | 2.020 |  | 2.020 |  | 100.000 |  |
|  |  | Fehlend |  | 0 |  | 0.000 |  |  |  |  |  |
|  |  | Gesamt |  | 1634 |  | 100.000 |  |  |  |  |  |
| Moderat (4-7) |  | Nein |  | 1972 |  | 97.915 |  | 97.915 |  | 97.915 |  |
|  |  | Ja |  | 42 |  | 2.085 |  | 2.085 |  | 100.000 |  |
|  |  | Fehlend |  | 0 |  | 0.000 |  |  |  |  |  |
|  |  | Gesamt |  | 2014 |  | 100.000 |  |  |  |  |  |
| Stark (8-10) |  | Nein |  | 581 |  | 96.672 |  | 96.672 |  | 96.672 |  |
|  |  | Ja |  | 20 |  | 3.328 |  | 3.328 |  | 100.000 |  |
|  |  | Fehlend |  | 0 |  | 0.000 |  |  |  |  |  |
|  |  | Gesamt |  | 601 |  | 100.000 |  |  |  |  |  |
|  | | | | | | | | | | | |

| Häufigkeiten für NA-Notwendigkeit nach EB Psyche | | | | | | | | | | | |
| --- | --- | --- | --- | --- | --- | --- | --- | --- | --- | --- | --- |
| EBr2-Psyche | | NA-Notwendigkeit | | Häufigkeit | | Prozent | | Prozent gültig | | Prozent kumulativ | |
| unauffällig |  | Nein |  | 4333 |  | 98.321 |  | 98.321 |  | 98.321 |  |
|  |  | Ja |  | 74 |  | 1.679 |  | 1.679 |  | 100.000 |  |
|  |  | Fehlend |  | 0 |  | 0.000 |  |  |  |  |  |
|  |  | Gesamt |  | 4407 |  | 100.000 |  |  |  |  |  |
| nicht dokumentiert |  | Nein |  | 5164 |  | 97.214 |  | 97.214 |  | 97.214 |  |
|  |  | Ja |  | 148 |  | 2.786 |  | 2.786 |  | 100.000 |  |
|  |  | Fehlend |  | 0 |  | 0.000 |  |  |  |  |  |
|  |  | Gesamt |  | 5312 |  | 100.000 |  |  |  |  |  |
| Sonstiges |  | Nein |  | 1026 |  | 96.338 |  | 96.338 |  | 96.338 |  |
|  |  | Ja |  | 39 |  | 3.662 |  | 3.662 |  | 100.000 |  |
|  |  | Fehlend |  | 0 |  | 0.000 |  |  |  |  |  |
|  |  | Gesamt |  | 1065 |  | 100.000 |  |  |  |  |  |
| verwirrt |  | Nein |  | 44 |  | 89.796 |  | 89.796 |  | 89.796 |  |
|  |  | Ja |  | 5 |  | 10.204 |  | 10.204 |  | 100.000 |  |
|  |  | Fehlend |  | 0 |  | 0.000 |  |  |  |  |  |
|  |  | Gesamt |  | 49 |  | 100.000 |  |  |  |  |  |
|  | | | | | | | | | | | |

| Häufigkeiten für NA-Notwendigkeit nach Einsatzort | | | | | | | | | | | |
| --- | --- | --- | --- | --- | --- | --- | --- | --- | --- | --- | --- |
| Einsatzort r2 | | NA-Notwendigkeit | | Häufigkeit | | Prozent | | Prozent gültig | | Prozent kumulativ | |
| Wohnung |  | Nein |  | 3086 |  | 95.660 |  | 95.660 |  | 95.660 |  |
|  |  | Ja |  | 140 |  | 4.340 |  | 4.340 |  | 100.000 |  |
|  |  | Fehlend |  | 0 |  | 0.000 |  |  |  |  |  |
|  |  | Gesamt |  | 3226 |  | 100.000 |  |  |  |  |  |
| nicht dokumentiert |  | Nein |  | 6854 |  | 98.718 |  | 98.718 |  | 98.718 |  |
|  |  | Ja |  | 89 |  | 1.282 |  | 1.282 |  | 100.000 |  |
|  |  | Fehlend |  | 0 |  | 0.000 |  |  |  |  |  |
|  |  | Gesamt |  | 6943 |  | 100.000 |  |  |  |  |  |
| Arbeitsplatz |  | Nein |  | 141 |  | 95.270 |  | 95.270 |  | 95.270 |  |
|  |  | Ja |  | 7 |  | 4.730 |  | 4.730 |  | 100.000 |  |
|  |  | Fehlend |  | 0 |  | 0.000 |  |  |  |  |  |
|  |  | Gesamt |  | 148 |  | 100.000 |  |  |  |  |  |
| Pflegeeinrichtung |  | Nein |  | 179 |  | 92.268 |  | 92.268 |  | 92.268 |  |
|  |  | Ja |  | 15 |  | 7.732 |  | 7.732 |  | 100.000 |  |
|  |  | Fehlend |  | 0 |  | 0.000 |  |  |  |  |  |
|  |  | Gesamt |  | 194 |  | 100.000 |  |  |  |  |  |
| öffentlicher Raum |  | Nein |  | 200 |  | 94.787 |  | 94.787 |  | 94.787 |  |
|  |  | Ja |  | 11 |  | 5.213 |  | 5.213 |  | 100.000 |  |
|  |  | Fehlend |  | 0 |  | 0.000 |  |  |  |  |  |
|  |  | Gesamt |  | 211 |  | 100.000 |  |  |  |  |  |
| Sonstiges |  | Nein |  | 107 |  | 96.396 |  | 96.396 |  | 96.396 |  |
|  |  | Ja |  | 4 |  | 3.604 |  | 3.604 |  | 100.000 |  |
|  |  | Fehlend |  | 0 |  | 0.000 |  |  |  |  |  |
|  |  | Gesamt |  | 111 |  | 100.000 |  |  |  |  |  |
|  | | | | | | | | | | | |

# Deskription der Maßnahmen und verabreichten Medikamente

## Deskription der Maßnahmen

| Häufigkeiten für Atemwege freimachen | | | | | | | | | |
| --- | --- | --- | --- | --- | --- | --- | --- | --- | --- |
| ATEMW_FREI | | Häufigkeit | | Prozent | | Prozent gültig | | Prozent kumulativ | |
| J |  | 50 |  | 0.462 |  | 100.000 |  | 100.000 |  |
| Fehlend |  | 10783 |  | 99.538 |  |  |  |  |  |
| Gesamt |  | 10833 |  | 100.000 |  |  |  |  |  |

| Häufigkeiten für Entlastungspunktion | | | | | | | | | |
| --- | --- | --- | --- | --- | --- | --- | --- | --- | --- |
| ENTLAST_PU | | Häufigkeit | | Prozent | | Prozent gültig | | Prozent kumulativ | |
| Fehlend |  | 10833 |  | 100.000 |  |  |  |  |  |
| Gesamt |  | 10833 |  | 100.000 |  |  |  |  |  |

| Häufigkeiten für invasive Atemwegssicherung | | | | | | | | | |
| --- | --- | --- | --- | --- | --- | --- | --- | --- | --- |
| INTUBATION | | Häufigkeit | | Prozent | | Prozent gültig | | Prozent kumulativ | |
| J |  | 82 |  | 0.757 |  | 100.000 |  | 100.000 |  |
| Fehlend |  | 10751 |  | 99.243 |  |  |  |  |  |
| Gesamt |  | 10833 |  | 100.000 |  |  |  |  |  |

| Häufigkeiten für Sauerstoffgabe | | | | | | | | | |
| --- | --- | --- | --- | --- | --- | --- | --- | --- | --- |
| SAUERSTOFF | | Häufigkeit | | Prozent | | Prozent gültig | | Prozent kumulativ | |
| J |  | 638 |  | 5.889 |  | 100.000 |  | 100.000 |  |
| Fehlend |  | 10195 |  | 94.111 |  |  |  |  |  |
| Gesamt |  | 10833 |  | 100.000 |  |  |  |  |  |

| Häufigkeiten für Defibrillation | | | | | | | | | |
| --- | --- | --- | --- | --- | --- | --- | --- | --- | --- |
| DEFI | | Häufigkeit | | Prozent | | Prozent gültig | | Prozent kumulativ | |
| J |  | 36 |  | 0.332 |  | 100.000 |  | 100.000 |  |
| Fehlend |  | 10797 |  | 99.668 |  |  |  |  |  |
| Gesamt |  | 10833 |  | 100.000 |  |  |  |  |  |

| Häufigkeiten für Gefäßzugang | | | | | | | | | |
| --- | --- | --- | --- | --- | --- | --- | --- | --- | --- |
| ZUGANG | | Häufigkeit | | Prozent | | Prozent gültig | | Prozent kumulativ | |
| J |  | 3518 |  | 32.475 |  | 100.000 |  | 100.000 |  |
| Fehlend |  | 7315 |  | 67.525 |  |  |  |  |  |
| Gesamt |  | 10833 |  | 100.000 |  |  |  |  |  |

| Häufigkeiten für Kardioversion | | | | | | | | | |
| --- | --- | --- | --- | --- | --- | --- | --- | --- | --- |
| Spezielle Maßnahmen - Kardioversion | | Häufigkeit | | Prozent | | Prozent gültig | | Prozent kumulativ | |
| Fehlend |  | 10833 |  | 100.000 |  |  |  |  |  |
| Gesamt |  | 10833 |  | 100.000 |  |  |  |  |  |

| Häufigkeiten für Pacer/Schrittmacher | | | | | | | | | |
| --- | --- | --- | --- | --- | --- | --- | --- | --- | --- |
| Pacer/Schrittmacher | | Häufigkeit | | Prozent | | Prozent gültig | | Prozent kumulativ | |
| J |  | 3 |  | 0.028 |  | 100.000 |  | 100.000 |  |
| Fehlend |  | 10830 |  | 99.972 |  |  |  |  |  |
| Gesamt |  | 10833 |  | 100.000 |  |  |  |  |  |
|  | | | | | | | | | |

## Deskription der Medikamente

|  | | Gültig | |
| --- | --- | --- | --- |
| ASS |  | 3818 |  |
| ADENOSIN |  | 4 |  |
| ADRENALI |  | 70 |  |
| AKRINOR |  | 82 |  |
| AMIODARO |  | 55 |  |
| ANTIBIOS |  | 3 |  |
| ATROPIN |  | 42 |  |
| BETABLOC |  | 404 |  |
| BUSCOPAN |  | 4 |  |
| CALCIUM |  | 5 |  |
| CLONIDIN |  | 1 |  |
| CORTISON |  | 35 |  |
| DIGOXIN |  | 4 |  |
| DIMENHYD |  | 245 |  |
| DIMETIDE |  | 1 |  |
| DOBUTAMI/Dopamin |  | 8 |  |
| EK |  | 1 |  |
| EBRANTIL |  | 451 |  |
| ETOMIDAT |  | 0 |  |
| FENTANYL |  | 39 |  |
| FLUMACEN |  | 1 |  |
| FUROSEMI |  | 96 |  |
| GLUCOSE |  | 11 |  |
| GLYCEROL |  | 635 |  |
| ONDANSET |  | 734 |  |
| HEPARIN |  | 3168 |  |
| IPRATROP |  | 35 |  |
| KALIUM |  | 6 |  |
| LORAZEPA |  | 25 |  |
| LYSE |  | 3 |  |
| MAGNESIU |  | 49 |  |
| METAMIZO |  | 48 |  |
| METOCLOP |  | 22 |  |
| MIDAZOLA |  | 105 |  |
| MORPHIN |  | 1701 |  |
| NALOXON |  | 1 |  |
| NATRIUMB |  | 5 |  |
| NITRENTI |  | 0 |  |
| NORADREN |  | 48 |  |
| ORCIPREN |  | 0 |  |
| PANCURON |  | 3 |  |
| PANTOPRA |  | 6 |  |
| PARACETA |  | 6 |  |
| PHENHYDA |  | 1 |  |
| PIRITRAM |  | 7 |  |
| PROPOFOL |  | 49 |  |
| ROCURONI |  | 22 |  |
| SALBUTAM |  | 69 |  |
| SIMVASTA |  | 0 |  |
| SKETAMIN |  | 12 |  |
| SUCCINYL |  | 5 |  |
| SUFENTAI |  | 39 |  |
| TERBUTAL |  | 4 |  |
| THEOPHYL |  | 1 |  |
| VEL |  | 5675 |  |
|  | | | |

# Zuordnung von Medikamenten und Maßnahmen zum Pyramidenprozess

Die Zuordnung von Medikamenten und Maßnahmen erfolgte anhand dieser Publikationen:

Bundesverband der Ärztlichen Leitungen Rettungsdienst Deutschland. (2024). Medikamentenkatalog „heilkundliche Maßnahmen durch Notfallsanitäterinnen und -sanitäter“. <https://www.bv-aelrd.de/mod/resource/view.php?id=396>

Bundesverband der Ärztlichen Leitungen Rettungsdienst Deutschland. (2024). Maßnahmenkatalog „heilkundliche Maßnahmen durch Notfallsanitäterinnen und -sanitäter“. <https://www.bv-aelrd.de/mod/resource/view.php?id=395>

Deutscher Berufsverband Rettungsdienst. (2025). Musteralgorithmen 2025 zur Umsetzung des Pyramidenprozesses im Rahmen des NotSanG. <https://www.dbrd.de/images/algorithmen/DBRDAlgo25_V10_WEB2.pdf>

## Zuordnung der Maßnahmen zum Pyramidenprozess

| Maßnahme | Pyramiden-prozess | Indikation |
| --- | --- | --- |
| Atemweg freimachen | Ja | Eskalierendes Atemwegsmanagement |
| Entlastungspunktion | Ja | Spannungspeunmothorax |
| Intubation | Nein^[1]^ |  |
| Sauerstoffgabe | Ja | Eskalierendes Atemwegsmanagement |
| Defibrillation | Ja | Reanimation |
| NIV | Ja | Eskalierendes Atemwegsmanagement |
| Kardioversion | Ja | Notfallkardioversion |
| Pacer | Ja | Bradykardie |

## Zuordnung der Medikamente zum Pyramidenprozess

| Medikament aus den Protokollen | Pyramiden- prozess | Indikation |
| --- | --- | --- |
| ASS | J | ACS |
| Adenosin | Nein |  |
| Adrenalin | Ja | Bradykardie, Anaphylaxie, Pseudokrupp |
| Akrinor | Nein |  |
| Amiodaron | Ja | Notfallkardioversion |
| Antibiose | Nein |  |
| Atropin | Ja | Bradycardie, Alkylphosphatintoxikation |
| Betablocker | Ja | ACS |
| Butylscopolamin | Ja | Schmerzen/Koliken |
| Calcium | Nein |  |
| Clonidin | Nein |  |
| Cortison | Ja | Bronchoobstruktion, Anaphylaxie, Pseudokrupp |
| Digoxin | Nein |  |
| Dimenhydrinat | Ja | Massive Übelkeit/Erbrechen |
| Dimetinden | Ja | Anaphylaxie |
| Dopamin, Dobutamin | Nein |  |
| EK | Nein |  |
| Urapidil | Ja | Hypertensiver Notfall |
| Etomidate | Nein |  |
| Fentanyl | Ja | Starke Schmerzen |
| Flumacenil | Nein |  |
| Furosemid | Ja | Kardiales Lungenödem |
| Glucose | Ja | Verdünnung Amiodaron, Hypoglykämie |
| Glycerolnitrat | Ja | ACS, Kardiales Lungenödem |
| Ondansetron | Ja | Opiatgabe, Massive Übelkeit/Erbrechen |
| Heparin | Ja | ACS, Lungenembolie |
| Ipratropiumbromid | Ja | Bronchoobstruktion |
| Kalium | Nein |  |
| Lorazepam | Nein |  |
| Lyse | Nein |  |
| Magnesium | Nein |  |
| Metamizol | Ja | Schmerzen/Koliken |
| Metoclopramid | Nein^[2]^ |  |
| Midazolam | Ja | ACS, Krampfanfall, Starker Erregungszustand, Analgosedierung (i. V. m. Esketamin) |
| Morphin | Ja | ACS |
| Naloxon | Ja | Opiatintoxikation |
| Natriumbicarbonat | Nein |  |
| Nitrentipin | Ja | Hypertensiver Notfall |
| Noradrenalin | Nein |  |
| Orciprenalin | Nein |  |
| Pancuronium | Nein |  |
| Pantoprazol | Nein |  |
| Paracetamol | Ja (supp) | Fieberkrampf, päd. Schmerzen |
| Piritramid | Nein^[3]^ |  |
| Propofol | Nein |  |
| Rocuronium | Nein |  |
| Salbutamol | Ja | Bronchoobstruktion |
| Simvastatin | Nein |  |
| S-Ketamin | Ja | Starke Schmerzen |
| Succinylcholin | Nein |  |
| Sufentanil | Ja | Starke Schmerzen |
| Terbutalin | Nein |  |
| Theophyllin | Nein |  |
| VEL | Ja | Ohne genaue Angaben |

1. Die Daten erlauben keine Differenzierung zwischen den verschiedenen Atemwegssicherungsdevices. Im. Sinne der Beantwortung der Forschungsfrage wird dies als unerheblich eingeschätzt, da eine invasive Atemwegssicherung durch NotSan nicht ohne Hinzuziehung einer Notärzt:in erfolgt.

   Davon unbenommen liegen die entsprechenden Techniken im Kompetenzbereich von NotSan und sind auch im Pyramidenprozess enthalten.
2. Veraltet, andere Antiemetika (z.B. Dimenhydrinat) sind im Pyramidenprozess enthalten
3. Nicht im Pyramidenprozess enthalten, jedoch als NotSan-Maßnahme gewertet, da Piritramid problemlos durch andere, im Pyramidenprozess enthaltende Substanzen ersetzt werden könnte.
